# The Efficacy of Twin-Block Appliances for the Treatment of Obstructive Sleep Apnea in Children: A Systematic Review and Meta-Analysis

**DOI:** 10.1155/2022/3594162

**Published:** 2022-07-11

**Authors:** Jun Duan, Wanyuan Xia, Kai Yang, Xuelei Li, Feng Zhang, Jie Xu, Ying Jiang, Jia Liang, Bing Li

**Affiliations:** ^1^Department of Stomatology, Children's Hospital of Chongqing Medical University, National Clinical Research Center for Child Health and Disorders, Ministry of Education Key Laboratory of Child Development and Disorders, Chongqing Key Laboratory of Pediatrics, 400014, China; ^2^Department of Public Health and Management, Chongqing Three Gorges Medical College, Wanzhou, Chongqing 404120, China; ^3^The First Affiliated Hospital of Chongqing Medical University, No. 1 Youyi Road, Yuzhong District, Chongqing 400016, China; ^4^Department of Otolaryngology, Children's Hospital of Chongqing Medical University, 400014, China

## Abstract

**Objective:**

To evaluate the efficacy of twin-block appliance in the treatment of children with obstructive sleep apnea (OSA).

**Methods:**

Two independent reviewers conducted a systematic review of seven databases from database establishment until October 16, 2021. There were no language restrictions. The outcomes were changes in apnea-hypopnea index (AHI), oxyhemoglobin desaturation index (ODI), and lowest arterial oxygen saturation (lowest SaO_2_). National Institute for Health and Clinical Excellence (NICE) tool was used to assess the quality of the studies included.

**Results:**

A total of 207 articles were screened for relevance, and 6 of them met the inclusion criteria for our meta-analysis. Four of the studies were case series, 1 was nonrandomized control trial, and 1 was a randomized crossover clinical trial. After twin-block therapy, there was a significant decrease in AHI (4.35 events/hour, 95% CI: 4.04, 4.66, *p* ≤ 0.001). The lowest SaO_2_ significantly increased by 9.17% (95% CI: 12.05, 6.28, *p* ≤ 0.001). Sensitivity analysis by excluding studies one by one showed stable and favorable results in lowest SaO_2_ and AHI.

**Conclusions:**

Results from the meta-analysis showed that the use of twin-block appliance significantly decreased AHI and significantly increased lowest SaO_2_. Hence, twin-block appliance therapy may be an effective method for the treatment of pediatric OSA. Further large sample size randomized controlled trials are needed to assess this treatment efficacy in children with obstructive sleep apnea.

## 1. Introduction

Obstructive sleep apnea (OSA) is a sleep disorder in which complete or partial upper airway obstruction occurs repeatedly during sleep, resulting in frequent apneas or reduced ventilation [1]. The prevalence of childhood OSA is high and can occur in children of every age, from newborn to adolescents. The 2012 American Academy of Pediatrics guidelines reported that the prevalence of OSA in children is between 1.2% and 5.7% [2]. In 2010, Hong Kong, China, reported a pediatric OSA prevalence of 4.8% [3]. Causes of OSA in children include adenotonsillar hypertrophy, neuromuscular disease, obesity, and craniofacial abnormalities [4]. Pediatric OSA has a peculiar clinical, diagnostic criteria, and treatment. It is an independent clinical syndrome with hidden manifestation onset and can cause great harm. Partial or complete upper airway obstruction during sleep in children with OSA leads to decreased blood oxygen saturation and high blood carbon dioxide content, which consequently results in cognitive impairment, excessive daytime sleepiness, and attention deficit. It can also lead to emotional instability and an increased risk of depression. Accordingly, it affects the health of children and leads to the emergence of pediatric growth and development problems [5–7]. Additionally, OSA in children can manifest as dentition problems, protruding anterior teeth, upper arch stenosis, underdevelopment of the mandible, underdevelopment of the chin, high arch of the hard palate, night snoring, mouth breathing, nocturia, night terrors, headaches [8], etc. [9].

Presently, there is a relative consensus on the treatment of adult OSA, but the choice of OSA treatment in children is still controversial. Commonly used clinical treatments for pediatric OSA include adenoid tonsillectomy, continuous positive airway pressure (CPAP), oral appliance (OA), and medications [10]. The choice and effectiveness of these treatment options are dependent on the causes of airway obstruction, the severity of airway obstruction, and patient compliance [11]. Maintaining positive pressure ventilation of air passages requires good compliance by patients. Children are growing, and long-term use of CPAP can easily cause irreversible changes to the maxillofacial region [12].

In view of the risk factors of adenotonsillar hypertrophy in children with OSA, anti-inflammatory drugs have been proposed as a potential nonsurgical treatment option. However, the long-term efficacy and safety of anti-inflammatory drugs in children with OSA is unknown [13].

A short-term retrospective study of 400 preadolescent children with sleep-disordered breathing (SDB), 3 months after the surgical intervention, concluded that SDB is involved in upper airway obstruction, which may be partly due to craniofacial involvement and that adenoidectomy may not be feasible for some patients because it may result in postoperative residual problems [14]. In addition, patient-centered outcomes such as concentration ability, alertness, or school performance have not been investigated [13].

Considering that the craniofacial involvement of OSA in children is common and the common clinical manifestations are retraction of the jaw and Angle Class II malocclusion, mandibular prefrontal devices can be used clinically for these patients, including Twin-block, Frankel II, Activator, and Herbst appliance [15]. Twin-block is a removable oral functional appliance designed by Clark and commonly used in orthodontic clinics for mandibular hypoplasia [16]. The appliance is a bite block that effectively modifies the bite ramp to induce a clockwise bite force by causing a functional mandibular displacement. The upper and lower occlusal blocks interlock at a 45-degree angle and are designed for all-weather wear to take full advantage of all the power applied to the dentition, including chewing power. The patient can eat comfortably after wearing the bite. When children with OSA wear the appliance, the jaw is forced to be extended forward, driving the tongue away from the pharynx and reducing the backward collapse of the tongue. It may improve the patency of the upper respiratory tract and relieve obstruction by changing the position of the hyoid bone or the size of the airway.

As of the period, the Cochrane systematic review was published in 2016, and there was limited high-quality evidence to affirm the effectiveness of OAs in the treatment of pediatric OSA [17]. Since then, few articles on the efficacy of these appliances have been published. However, the reports on the efficacy of the twin-block appliance are inconsistent and unconvincing. Existing studies on twin-block treatment of OSA rarely use other orthotics and treatment methods as controls, and they mainly use their own pre- and postcontrol. Most of the existing studies reported that twin-block had some effect on OSA, but the sample size was small. We do not know if a larger sample size would show the same results.

Thus, we conducted a systematic review and meta-analysis of these studies. In order to provide a reference for orthodontic clinical practice, this study reviewed literatures on twin-block appliance treatment of OSA in children, summarized the current Cochrane systematic review evidence for orthodontic treatment in children, and evaluated the methodological quality of the included studies.

## 2. Methods

### 2.1. Selection Criteria

Two reviewers (Wanyuan Xia and Jun Duan) independently screened the articles for inclusion and performed the data extraction. Studies available as abstracts only were included if we could verify the inclusion and exclusion criteria, and at least one of the outcomes of interest was reported. Each article was reviewed by the two independent reviewers using the following standardized inclusion criteria: (1) children below 18 years and diagnosed as having obstructive sleep apnea syndrome (OSAS) or obstructive sleep apnea hypopnea syndrome (OSAHS), (2) underwent twin-block or modified twin-block appliance treatment, (3) available pre- and postintervention sleep study data, (4) outcomes were reported as the improvement in at least one of the three overnight in laboratory polysomnography measurements (apnea-hypopnea index (AHI), oxyhemoglobin desaturation index (ODI), and lowest arterial oxygen saturation (Lowest SaO_2_)), and (5) study design was either case series, case-control, cohort, retrospective controlled trials, and/or randomized controlled trials (RCTs). The exclusion criteria were as follows: (1) studies with participants who are older than 18 years; (2) studies that did not provide quantitative data; (3) studies that did not use twin-block or modified twin-block appliances as a treatment for OSA; (4) research that was case report, animal experiments, comments or review; and (5) the total sample size was less than 10. Discrepancies during abstract and full-text screening were resolved by discussion with each other and consultation with a third reviewer (Feng Zhang) until consensus was reached.

### 2.2. Database and Search Strategy

We searched seven databases, namely, PubMed, Embase, the Cochrane Library, Chinese Biomedical Database (CBM), Chinese National Knowledge Infrastructure, VIP Database, and Wanfang, from database establishment to October 16, 2021, using keywords like “Child,” “pediatrics,” “Adolescent,” “Sleep Apnea Syndromes,” “Sleep Apnea,” “Obstructive,” and “twin block.” The detailed search strategy on PubMed is shown in supplementary materials as a retrieval example table s1. Reference lists of included studies were handsearched to identify additional relevant literature.

### 2.3. Data Extraction and Quality Assessment

Each literature was screened based on the inclusion criteria, and the data from the included studies were extracted by two independent researchers. Using the inclusion criteria, first author, publication year, research types, sample size, mean age, gender, diagnostic criteria, treatment duration, interventions, follow-up time, and outcome indicators were extracted from the studies. The quality of the studies was assessed using the National Institute for Health and Clinical Excellence (NICE) tool [18](total 8 items, quality assessment score ≥ 5 was definite as high-quality study, and quality assessment score < 5was definite as low-quality study.). The overall quality assessment was based on an independent evaluation by two reviewers, and in case of discrepancies, discussions inclusive of a third reviewer were held, until consensus was reached.

### 2.4. Statistical Methods

The Preferred Reporting Items for Systematic Reviews and Meta-Analysis (PRISMA) guidelines were utilized for this research as far as possible [19]. Mean and standard deviation were calculated before and after twin-block therapy for AHI, mean oxygen saturation, and Lowest SaO_2_. The null hypothesis for this study is that there is no difference in the outcome data before and after twin-block therapy. R software version 4.1.1 was used for the meta-analysis, and a random effect model was used throughout the analysis. The mean, standard deviation, and 95% confidence interval (CI) were calculated. *I*^2^ statistic was used to determine the level of inconsistency (low = 25%, moderate = 50%, and high = 75%). Cochran *Q* statistic was used to determine heterogeneity, with a *p* value ≤ 0.1 considered significant heterogeneity [20]. If inconsistency and/or heterogeneity was identified, a sensitivity analysis was performed by changing the combined model (fixed effects model and random effects model) and individually removing one study at a time. Forest plots were created after extracting pre- and post-twin-block therapy data for each of the primary outcomes. Mean difference and effect estimate were combined using random effects meta-analysis for AHI, mean oxygen saturation, and Lowest SaO_2_. Effect estimate was reported for all three outcomes. Publication bias was assessed by Egger's test, Begg's test, and funnel.

## 3. Results

**Figure 1:**
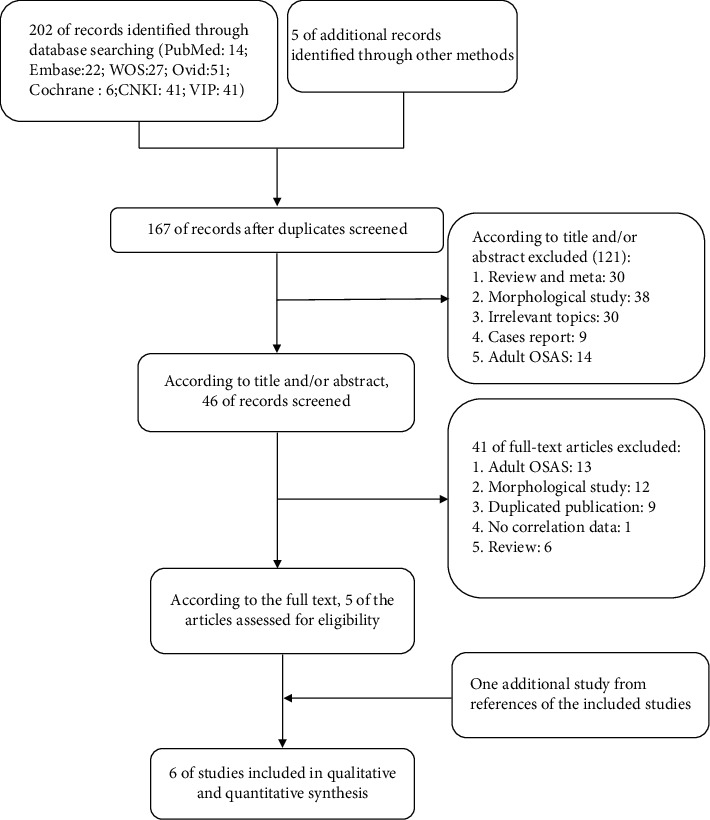
Literature screening flow.

A total of 207 articles were screened for relevance. Only 6 of them met the inclusion criteria for our meta-analysis (Figure 1), 4 of the studies were case series, 1 was nonrandomized control trial, and 1 was a randomized crossover clinical trial. The total sample size of the 6 articles was 170, with a mean age of 11.36 years and 59.4% male participants (Table 1, Table S2). The result of Egger's test (*p* = 0.1456) and Begg's test (*p* = 0.8806) reveals that there is not publish bias in including the article, and the funnel in the supplement materials (figure s3).

**Figure 2:**
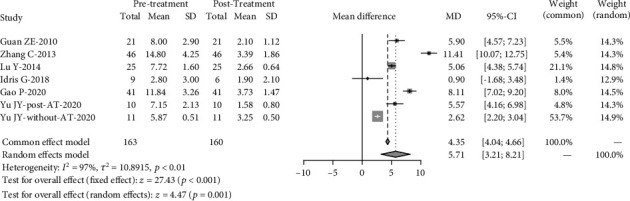
Comparison of apnea-hypopnea index (AHI) before and after twin-block treatment. AT: adenotonsillectomy.

**Figure 3:**
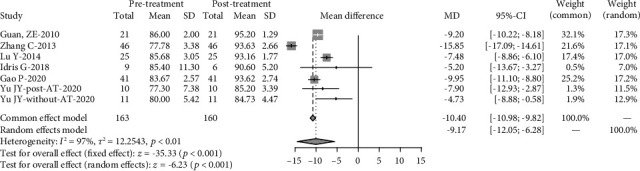
Comparison of lowest arterial oxygen saturation (Lowest SaO_2_) before and after twin-block treatment. AT: adenotonsillectomy.

After twin-block therapy, there was a significant decrease in AHI (4.35 events/hour, 95% CI: 4.04, 4.66, *p* ≤ 0.001). Both the *I*^2^ (97%) and *Q* statistics (*p* ≤ 0.001) indicated significant heterogeneity (Figure 2). Lowest SaO_2_ significantly increased by 9.17% (95% CI: 12.05, 6.28), with a significant heterogeneity (*I*^2=^94%) and (*Q* statistics, *p* ≤ 0.001) (Figure 3). There was no significant increase in mean oxygen saturations, with no significant heterogeneity (*I*^2^ = 0%, *Q* statistics, *p* = 0.79) (Figure s1).

**Figure 4:**
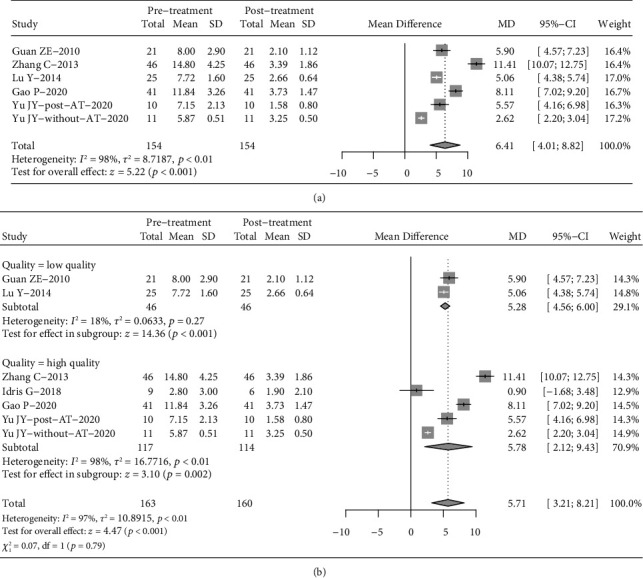
Pooling for apnea-hypopnea index (AHI) (a) and lowest arterial oxygen saturation (Lowest SaO_2_) (b), which patients were definitely diagnosed as mandibular retraction. AT: adenotonsillectomy.

**Figure 5:**
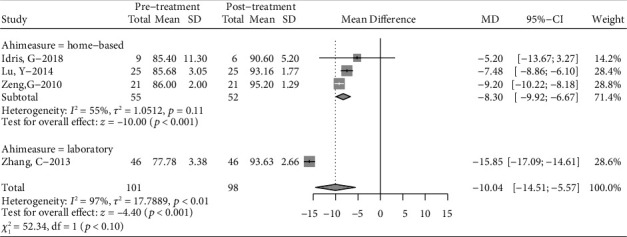
Subgroup analysis for lowest arterial oxygen saturation (Lowest SaO_2_) according to AHI measure location. AT: adenotonsillectomy.

Considering the high heterogeneity of the meta-analysis, subgroup analysis was conducted according to the severity of AHI, type of appliance, literature quality, and treatment duration. High heterogeneity of subgroup differences was observed for literature quality, with no significant subgroup difference (Figure S2). We excluding the article included Angle Class I participation and pooling the rest article. The pooling result reveal that AHI and Lowest SaO_2_ still have a significant improvement (Figure 4), suggesting that long-term treatment (more than 12 months) may be more effective. A subgroup meta-analysis was also performed for Lowest SaO_2_ based on the OSA testing site (home vs. laboratory) (Figure 5). The combined results showed that there was a statistically significant difference in the twin-block treatment between the unsupervised home testing/monitoring group (mean difference = 7.82, 95% CI: (6.20, 9.44), *p* = 0.010) and the group tested/monitored in the laboratory (mean difference = 12.90, 95% CI: (7.11, 18.68), *p* ≤ 0.001) (Figure 5). The results indicate that Lowest SaO_2_ was effective mitigation in each subgroup.

**Figure 6:**
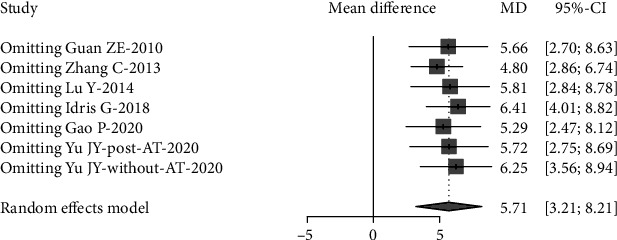
Sensitivity analyses of apnea-hypopnea index (AHI), iteratively removing each study from the overall analysis. AT: adenotonsillectomy.

**Figure 7:**
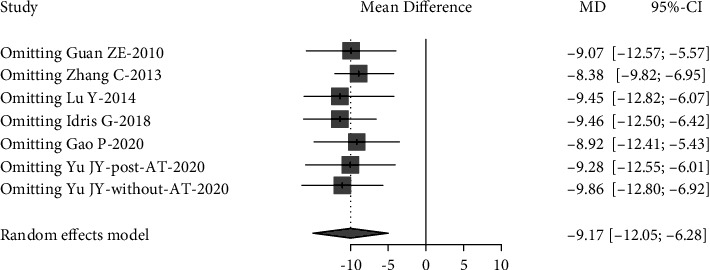
Sensitivity analyses of Lowest SaO_2_, iteratively removing each study from the overall analysis. AT: adenotonsillectomy.

Sensitivity analysis was performed to investigate the influence of individual studies on the Lowest SaO_2_ and AHI treatment result (Figures 6 and 7). The result revealed that there was no reverse change in the meta-pooling. Changing the combined model reveal a stable result in AHI and LSaO_2_ (Figures 1 and 2).

## 4. Discussion

### 4.1. Summary of Main Results

This study performed a meta-analysis to comprehensively evaluate the effect of the twin-block appliance in the treatment of children with OSA. The use of a twin-block appliance resulted in a significant decrease in AHI and a significant increase in Lowest SaO_2_. The primary studies included in the meta-analysis had small sample sizes but we are interested in identifying a potential change or unstable outcome when the sample size is enlarged. The included studies [21–26] showed significant heterogeneity when AHI and Lowest SaO_2_ were merged. The heterogeneity of Lowest SaO_2_ significantly reduced when the analysis was stratified according to OSA measurement method (AHI measured at home: *I*^2^ = 48%, *p* = 0.1). The sensitivity analysis showed that the increase was stable for Lowest SaO_2_ and AHI. Mean oxygen desaturation increased but there was no significant difference in the two studies [27].

Compliance with twin-block appliance therapy for OSA treatment was good, as no patient abandoned the treatment except in Idris et al.'s study [21] where three patients abandoned the treatment. The high success rate in the included study might be because the subjects were selected from otherwise healthy OSA children with retrognathia mainly. Other factors such as adenotonsillar hypertrophy or obesity causing OSA were excluded in the eligibility criteria except Yu's study. Hence, it needed more high-quality evidence to confirm the conclusion that oral instruments and functional orthopedic instruments are effective in the treatment of obstructive sleep apnea in children, which is consistent with Carvalho et al.'s research [17].

Cephalometric measurements are often used to describe the outcome of orthodontic treatment. For example, Idris et al. used cephalometric measurement to describe the dentofacial features of participants. Cephalometric measurements in Zhang's study showed significant increase in upper and posterior airway space, intermediate airway space, SNB angle, and facial protruding, indicating enhanced mandibular growth.

### 4.2. AHI Evaluation Criteria and PSG Monitor Environment

The main cause of OSA in children is adenoid and/or tonsillar hypertrophy. If the degree of gland hypertrophy is different, the severity of OSA is also different. The amount of reduction would be considered clinically significant according to one of the common definitions of successful OSA treatment (a reduction of ≥50% in AHI) and another criterion for complete resolution of OSA symptoms (AHI reduction to less than one event/h) [28, 29]. Except the study by Idris et al., [21] which reported successful OSA treatment and complete resolution of OSA symptoms, other studies did not report on these indicators. Thus, future studies should report the indicators of successful OSA treatment and complete resolution of OSA symptoms.

Moreover, based on the wishes of parents, in the context of children who are unable to go to the hospital for polysomnography, we consider home polysomnography. This time, it was done as an alternative. However, the inconsistency of polysomnography indicators will affect the evaluation of results; hence, the need for a unified polysomnography instrument and environment is pertinent. We carry out an analysis of subgroup between home-based measure and laboratory measure. And the result found there are no significant difference of therapy efficacy between two groups.

### 4.3. Therapy Efficacy

The gold standard for diagnosing OSA is overnight in laboratory polysomnography. According to the American Academy of Sleep Medicine. AHI ≤ 1 time/h is the benchmark while mild, moderate and severe AHI are 1 times/h < AHI ≤ 5 times/h, 5 times/h < AHI ≤ 10 times/h, and AHI > 10 times/h, respectively [2]. Diagnostic criteria for a child patient are different from those for an adult patient. Because OSA patients may have adenoid or tonsillar hypertrophy, obesity, different upper airway obstruction sites, and neuromuscular disorders, these factors may affect the AHI results. Future studies need to focus on the causes of OSA, develop treatment plans for the causes, and evaluate the therapeutic efficacy. For example, OA is used to treat OSA patients with cranial and maxillofacial abnormalities. If adenoids and/or tonsils are enlarged, surgery may be required to achieve stable results and improve children's symptoms with OSA.

The patients in Zhang's study wore it nearly 24 hours except for mealtime, Lu's and Yu's study asked patients to wear it throughout the day while Idris et al.'s study required patients to wear it overnight. Guan's and Gao's study did not describe when they were worn. The improved twin-block placed a spiral pedicle extender in the middle of the maxillary pad. Lu's and Yu's article used a modified twin-block, but the size of maxillary enlargement is not specified in the article. We conduct an analysis of subgroup between twin-block appliances and modified twin-block appliances. And the result found there are no significant difference of therapy efficacy between two groups. But, we still suggest that future studies will need to unify the duration of wear and the shape of the appliance to enhance the comparability of studies.

The SNB range of the subjects included in Idris's paper was 70.0°–82.0°, and the ANB range was 0.5°–7.0°, so this paper included the subjects of Angle Class II mandibular retraction and Angle Class I. Zhang's article included patients with mandibular retraction of ANB > 3° and SNB < 80°; Guan's study incorporated patients with mandibular retraction of ANB > 5°. In Yu's study, patients with mandibular retraction of ANB > 4° and SNB < 78° were included. Lu's study included Angle Class II patients with mandibular retraction. Gao's study included patients with early permanent dentition with mandibular retraction. Except for Idris's paper, the remaining 5 articles included OSA patients with mandible retraction. The pooling result reveal that there were significant decrease in AHI and significant increase in Lowest SaO_2_ after twin-block therapy.

The degree of mandibular retraction determines the degree of mandibular anterior lead and the degree of improvement of the total volume of the oropharyngeal air duct, the minimum cross-section area of the airway, the sagittal diameter of the minimum cross-section (mm), and the transverse diameter of the minimum cross-section (mm). Future studies may explore the correlation between the degree of changes in SNB, ANB, and anterior tooth overjet after mandibular forward movement and the degree of improvement in airway space and AHI.

### 4.4. Appliance Selection

At present, the clinical oral appliances for mandibular retrognathia in children include twin-block, Frankel 2 (FR2), Activator, Herbst, and Bionator [30]. The manufacture of Herbst appliance is complicated. Compared with FR2, Activator, and Bionator, twin-block can be worn all day long even when eating, exercising, or speaking. It has the characteristics of simple fabrication, convenient wearing, wide indications, and high efficacy, so it is widely used in clinical orthodontic treatment [31]. Some studies failed to mention which type of appliance used. Different appliances have their own characteristics and treatment effects and the treatment effects of different oral appliances in children with OSA were not completely unified. Hence, future research should explore the therapeutic effects of different appliances on children with OSA, vis-a-vis other influencing factors such as patient's age and time of wearing oral appliance.

Four systematic reviews [17, 29, 32, 33] were previously published on mandibular advancement appliances (MAA) treatment for pediatric OSA but none of them focused on twin-block appliances. Few studies on children with OSA describe the efficacy in different types of MAA. Additionally, some of the studies on twin-block appliance were published in Chinese. These previous studies did not assess the efficacy of the different types of MAA. Our study is the first to synthesize studies on twin-block appliance in order to estimate its efficacy.

### 4.5. About Cephalometric Techniques

Cephalometric analysis was introduced into orthodontics in 1931 [34]. In 1994, some scholars established an airway measurement program [35]. Since many children with OSA have narrow airways [36], cephalometric measurement also has great advantages in assessing changes in airway cross-sectional area and volume before and after treatment [37]. In the included articles, the article by Lu analyzed the 3d airway cone-beam computed tomography reconstruction image of the children before and after treatment and showed that the total volume of oropharyngeal airway, minimum airway cross-sectional area, minimum sagittal diameter, and minimum cross-section diameter of the children increased after functional correction compared with before treatment. Guan's study showed that the ANB angle increased in children with OSA after orthodontic treatment, indicating an increase in mandibular length or anterior mandibular position. Cephalometric measurements in Yu's study and Gao's study showed that the mandibular retraction type of children with OSA was improved after treatment. Hence, cephalometry is an important tool for the assessment of cranial and maxillofacial soft and hard tissues, which needs more attention.

## 5. Limitations

Our study had some limitations. Firstly, only one RCT was included in the primary meta-analysis and the sample size was small. Hence, more well-designed RCTs with large-sample sizes are needed. Secondly, only one research assessed the side effect of MAA, and other studies did not assess their side effect. Thirdly, the studies were restricted to the most frequently used the AHI value, and only two studies involved other important clinical outcomes like quality of life and sleep structure. Fourthly, we did not include craniofacial development in the quantitative pool and there was no research to compare other functional appliances or therapy methods. Lastly, all the studies were conducted before puberty; hence, the effect of the treatment during puberty is unknown.

## 6. Conclusions

Our systematic review found that twin-block appliance in the treatment of pediatric OSA patients was associated with a significant decrease in AHI and a significant increase in Lowest SaO_2_. Thus, twin-block appliance may be an effective method for treating pediatric OSA. Since the included studies are mainly, we are relatively prudent in drawing conclusions. Further randomized clinical studies are needed to assess the efficacy of this treatment approach in children with OSA. Furthermore, studies with increased quality through larger sample sizes and development of uniform inclusion and exclusion criteria are needed in the future. When studies on the use of twin-block appliance in the treatment of OSA in children and adolescents are reported through standardized data, the comparability of the studies based on the same outcome measurements will improve, which will help establish orthodontic treatment guidelines for children with OSA.

## Figures and Tables

**Table 1:** Characteristics of the included studies.

Study	Study design	Subgroup	Gender	Sample size	Age	Interventions	Eligibility criteria	Wearing time	Dropout	Primary outcomes	Secondary outcomes
Idris G	Crossover-RCT	Twin-block treatment	Male:13 Female:3	9	9.8 ± 1.4 (8 ~ 12)	Twin-block	Inclusion criteria: Age range from 8 to 12 years, and parental report of loud snoring for three or more nights per week. Exclusion criteria were: previous orthodontic treatment, craniofacial and genetic syndromes (e.g. cleft lip and/or palate), neuromuscular disorders, and class III incisor and/or skeletal relationship as confirmed by lateral cephalometric radiograph (ANB angle ≤0°)	3 weeks (overnight)	3	Apnea-hypopnea index, lowest oxygen saturation	Growth hormone levels, SDB symptoms and daytime sleepiness questionnaires, quality-of-life questionnaire (OSA-18), behavioral assessment (the behavioral and emotional screening system), parent report of nocturnal enuresis
		Control		9		Sham MAA			0		

Zhang C	Case series	Twin-block	Male: 31 Female:15	46	9.7 ± 1.5 ()	Twin-block	The inclusion criteria includes: Patients who were in category two or three of the cervical vertebrae maturation indices, which indicated that patients have not reached the peak pubertal growth spurt; patients who had mandibular retrognathia determined clinically as well as with the aid of cephalometric radiographs (ANB >3°; SNB < 80°; incisor overjet > 3 mm); patients who had snoring habit reported by their parents and an OSA (apnea/hypopnea index (AHI) > 1/h). The exclusion criteria included patients with acute upper airway infection, adenotonsillar hypertrophy, body mass index above cut off points of overweight which was announced by an international survey, orAHI ≤ 1/h were rejected from the study.	average10.8 months (24 H except for mealtime)	0	Apnea-hypopnea index, lowest oxygen saturation	Cephalometric measurements

Lu Y	Case series	Modified twin-block	Male: 14 Female:11	25	13.4 (12~15)	Modified twin-block	Inclusion criteria: (1) patients had no history of orthodontic treatment before treatment; (2) early permanent teeth, molar, cusp distal relationship; cephalometric showed vegetative form is the average angle or low angle, mainly with mandibular retraction angle ii class malocclusion; (3) in the early or peak period of growth and development; (4)PSG results showed that it met the diagnostic criteria of OSAHS in children; (5) no TMJ disease and adolescent periodontal disease; no inflammatory hyperplasia of tonsils or other anatomic factors that may cause OSAHS.	Average 12.7 months (all the time)	0	Apnea-hypopnea index, lowest oxygen saturation	Hard and soft tissues analysis, CBCT analysis of the upper airway

Guan ZE	Case series	Twin-block	Male: 14 Female:7	21	12.7 (10.4 ~ 14)	Twin-block	Not description	>1 year (not detail apnea-hypopnea index, lowest oxygen saturation led description)	0	Apnea-hypopnea index, lowest oxygen saturation	Cephalometric measurements

Gao P	Case series	Twin-block treatment	Male: 19 Female:22	41	12.5 ± 3.69 (12~15)	Twin-block	Inclusion criteria: (1) no history of orthodontic treatment; (2) on the maxillary development is normal, mandibular development is insufficient in patients with early permanent dentition; (3) in the early or peak period of growth and development; (4) long-term residents of Xining city, Qinghai Province (2260 meters above sea level) Exclusion criteria: (1) genetic diseases that can lead to asymmetric facial development; (2) a glandular body, rhinitis, tonsil disease or other anatomical causes of OSA-HS element; (3) the children could not cooperate or their guardians refused to participate in the study; (4) there are temporomandibular joint disease, adolescent periodontal disease, missing teeth, cleft lip and palate, facial partial trauma, unilateral condylar hypertrophy, tumor, etc.	6 ~ 12 month (not detailed description)	0	Apnea-hypopnea index, lowest oxygen saturation	Cephalometric measurements

Yu JY	Nonrandom control trial	Adenoid and/or tonsillectomy + modified twin-block treatment	Male: 5 Female: 5	10	10.3 ± 0.95	Adenoid and/or tonsillectomy+modified twin-block	(1) The patient had no history of orthodontic treatment before treatment; (2) clinical examination showed high arch of palatal cover, narrow upper dental arch and lower jaw “adenoid face”, such as retraction; (3) molars, cusps distal relationship, the following collar retraction based on angle CLASS II error ANB > 4°, SNB < 78°; (4) moderate and severe OSAS: portable polysomnography showed AHI25 and snoring, open-mouth breathing, lethargy, hyperactivity, inattention, and other clinical symptoms; (5) there are adenoids and/or tonsil hypertrophy: cephalic radiograph shows moderate to severe adenoid hypertrophy with A/N value > 0.6; press tongue to check almond body ID degree or above; (6)age 9 to 12 years old, mixed dentition or early permanent teeth; lateral cranial radiograph showed the patient bone age in CVMS II-CVMS III (early or peak growth and development of CVMS); body mass index (BMI). The value is below the overweight range; no obvious symptoms of temporomandibular arthropathy, n-o history of trauma, and no history of labial appointment, no history of orthognathic surgery or otorhinolaryngology	Average 373 days (all the time)	0	Apnea-hypopnea index, lowest oxygen saturation, obstructive apnea index	Quality-of-life questionnaire (OSA-18), cephalometric measurements, tonsillar oropharyngeal examination
		Simple modified twin-block treatment	Male: 5 Female: 6	11	10.18 ± 0.98	Modified twin-block		Average 406 days (all the time)	0		

^‡^Quality assessment score ≥ 5 was definite as high-quality study, and quality assessment score < 5 was definite as low-quality study. MAA: mandibular advancement appliances.

## Data Availability

The data used to support the findings of this study are included within the article.

## Supplementary Materials

Supplementary Materials Supplementary material files include three files. The file named “supplementary material” include “Table S1: search strategy in the PubMed.” “Table s2: quality appraisal of included literature.” “Figure S1: comparison of mean oxygen saturation before and after twin-block treatment.” “Figure S2: subgroup analysis for apnea-hypopnea index (AHI) according to literature quality.” Figure s3: trim funnel of including article. The file named “PRISMA-2020-Checklist” include a PRISMA checklist for check items for reporting in the meta-analysis. The file named “AMSTAR guideline” include AMSTAR checklist for assess the methodological quality of the meta-analysis.
