# Supplementary material for: The Efficacy of Twin-Block Appliances for the Treatment of Obstructive Sleep Apnea in Children: A Systematic Review and Meta-Analysis
Source: Biomed Res Int. 2022 Jul 11;2022:3594162. doi: 10.1155/2022/3594162 (PMC9293515; doi:10.1155/2022/3594162)
Supplement: Supplementary Materials — Supplementary material files include three files. The file named “supplementary material” include “Table S1: search strategy in the PubMed.” “Table s2: quality appraisal of included literature.” “Figure S1: comparison of mean oxygen saturation before and after twin-block treatment.” “Figure S2: subgroup analysis for apnea-hypopnea index (AHI) according to literature quality.” Figure s3: trim funnel of including article. The file named “PRISMA-2020-Checklist” include a PRISMA checklist for check items for reporting in the meta-analysis. The file named “AMSTAR guideline” include AMSTAR checklist for assess the methodological quality of the meta-analysis. [file 3594162.f1.zip › supplemental material (2).docx]

**supplemental material**

**Table S1.** **Search strategy in the PubMed**

| #1 | sleep apnea [Title/Abstract] |
| --- | --- |
| #2 | sleep apnoea [Title/Abstract] |
| #3 | Sleep disordered breathing [Title/Abstract] |
| #4 | "Sleep Apnea Syndromes"[ MeSH Terms] |
| #5 | "Sleep Apnea, obstructive"[ MeSH Terms] |
| #6 | #1 OR #2 OR #3 OR #4 OR #5 |
| #7 | twin-block [all fields] |
| #8 | twin-block [Title/Abstract] |
| #9 | Twin [Title/Abstract] |
| #10 | Block [Title/Abstract] |
| #11 | #9 AND #10 |
| #12 | twin [all fields] |
| #13 | block [all fields] |
| #14 | #12 AND #13 |
| #15 | #7 OR #8 OR #11 OR #14 |
| #16 | #6 AND #15 |

#1-#16Retrieval time: 16 October 2021.

**Table s2. Quality appraisal of included literature**

| appraisal Iterms | included literrature | | | | | |
| --- | --- | --- | --- | --- | --- | --- |
|  | Idris, G | Zhang, C | Lu, Y | Guan, ZE | Gao, P | Yu, JY |
| 1) Case series collected in more thanone center, i.e., multi-center study? | no | no | no | no | no | no |
| 2) Is the hypothesis/aim/objective of the study clearly described? | yes | yes | yes | yes | yes | yes |
| 3) Are the inclusion and exclusion criteria (case definition) clearly reported? | yes | yes | yes | no | yes | yes |
| 4) Is there a clear definition of the outcomes reported? | yes | yes | yes | no | yes | yes |
| 5) Were data collected prospectively? | yes | yes | no | no | no | no |
| 6) Is there an explicit statement that patients were recruited consecutively? | yes | no | yes | no | yes | yes |
| 7) Are the main findings of the study clearly described? | yes | no | no | no | yes | yes |
| 8) Are outcomes stratified (e.g., by abnormal results, disease stage, patient characteristics)? | yes | yes | no | yes | no | yes |
| total score | 7 | 5 | 4 | 2 | 5 | 6 |

**S1 Fig.** **Comparison of mean oxygen saturation before and after twin-block treatment.**


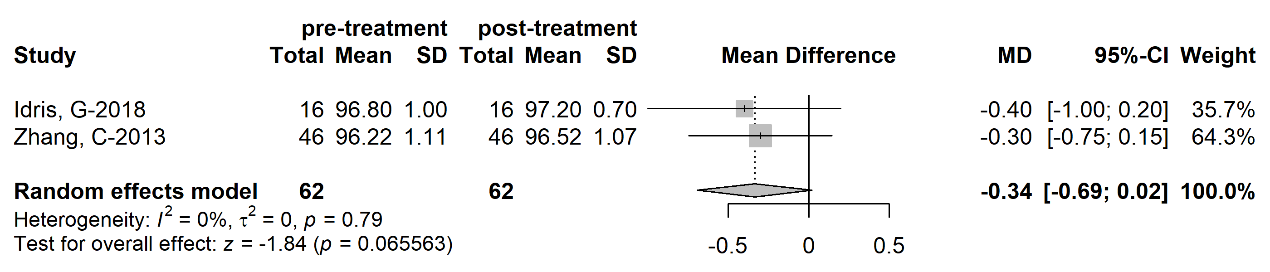


**S2 Fig. Subgroup analysis for apnea-hypopnea index (AHI) according to literature quality.** AT: adenotonsillectomy.


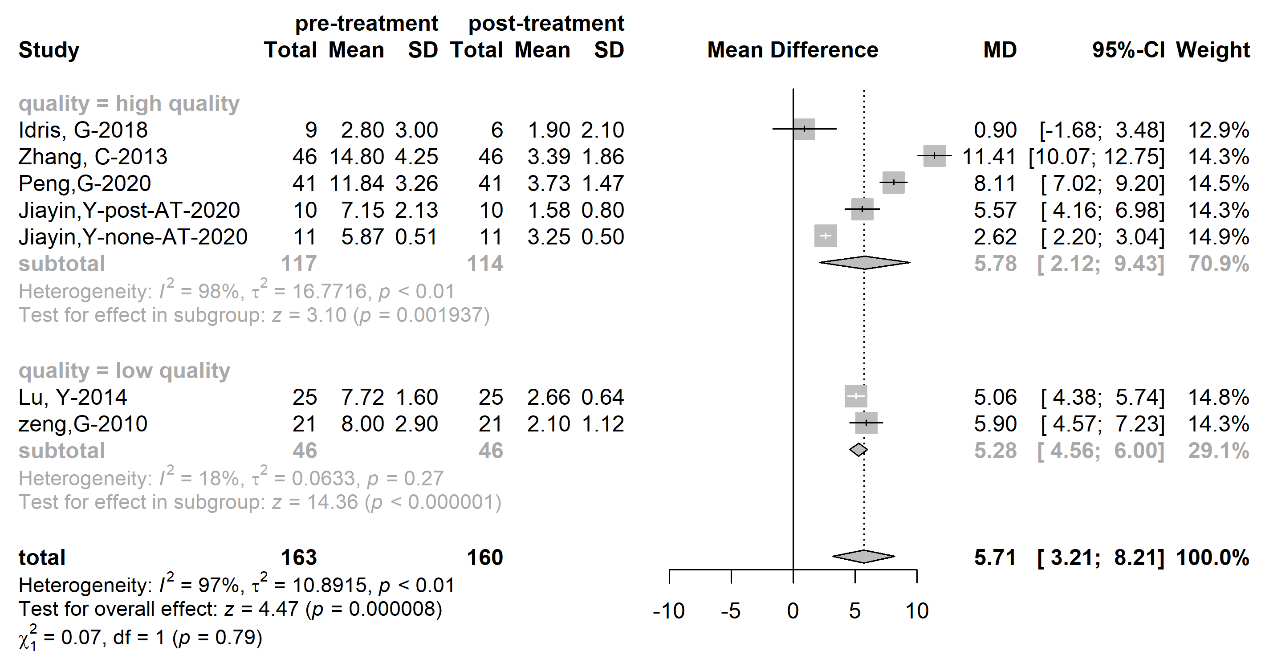


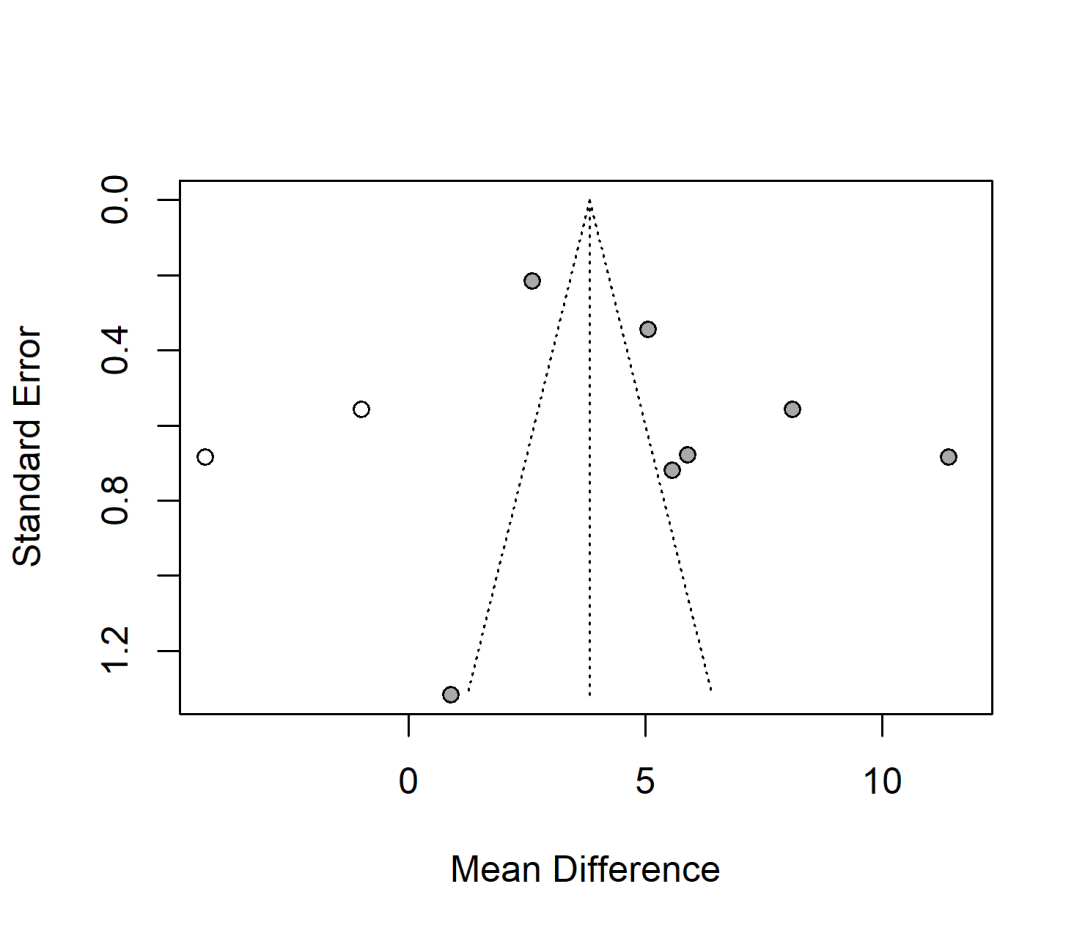


**figure s3: trimfunnel of including article**
